# The Neuromuscular Junction: Aging at the Crossroad between Nerves and Muscle

**DOI:** 10.3389/fnagi.2014.00208

**Published:** 2014-08-11

**Authors:** Marta Gonzalez-Freire, Rafael de Cabo, Stephanie A. Studenski, Luigi Ferrucci

**Affiliations:** ^1^Translational Gerontology Branch, National Institute on Aging, Intramural Research Program, National Institutes of Health, Baltimore, MD, USA; ^2^Longitudinal Studies Section, Baltimore Longitudinal Study of Aging, National Institute on Aging, National Institutes of Health, Baltimore, MD, USA

**Keywords:** aging, denervation, motor unit, neuromuscular junction, sarcopenia

## Abstract

Aging is associated with a progressive loss of muscle mass and strength and a decline in neurophysiological functions. Age-related neuromuscular junction (NMJ) plays a key role in musculoskeletal impairment that occurs with aging. However, whether changes in the NMJ precede or follow the decline of muscle mass and strength remains unresolved. Many factors such as mitochondrial dysfunction, oxidative stress, inflammation, changes in the innervation of muscle fibers, and mechanical properties of the motor units probably perform an important role in NMJ degeneration and muscle mass and strength decline in late life. This review addresses the primary events that might lead to NMJ dysfunction with aging, including studies on biomarkers, signaling pathways, and animal models. Interventions such as caloric restriction and exercise may positively affect the NMJ through this mechanism and attenuate the age-related progressive impairment in motor function.

## Introduction

Autopsy studies in persons who died of acute trauma while relatively healthy have shown that aging is associated with a gradual loss of motor neurons (Valdez et al., 2010; Rowan et al., 2012). The mechanism that leads to neuronal loss with aging is still unclear and may involve both impaired trophic signaling from the central nervous system (CNS), local degeneration, and feedback from dysfunctional muscle. Indeed, individuals over 65 years of age exhibit reduced activity in motor brain areas, increased oxidative stress in motor neurons and impaired energetic metabolism in muscle fibers (Deschenes et al., 2010; Ferrucci et al., 2012; Reid et al., 2012, 2014; Manini et al., 2013). However, there is evidence that this decrease in muscle energetic metabolism is not solely affected by aging but also by the levels of physical activity, suggesting that energetic changes are more likely to be a function of activity, and not time (Tevald et al., 2010; Russ and Lanza, 2011).

Regardless of the cause, when a motor neuron is lost, fibers previously innervated by that neuron, globally defined as a motor unit, are no longer controlled by the nervous system and fail to contribute to the force generated during a volitional muscle contraction. In the attempt to counteract the functional consequence of this process, denervated orphan fibers express proteins and produce chemotactic signals that stimulate the sprouting of new dendrites from residual motor neurons. This process leads re-innervation by the expansion of pre-existing motor units and is aimed at returning to function previously denervated muscle fibers. This dynamic denervation–re-innervation cycle successfully compensates for neuronal loss, with little decline in global strength and only slightly reduced control. However, there is evidence that this compensatory mechanism starts failing with aging. Some denervated fibers are not successfully re-innervated, become apoptotic, and are not replaced by new fibers. It is hypothesized that this phenomenon contributes to a progressive decline in muscle mass and strength with aging.

**Figure 1:**
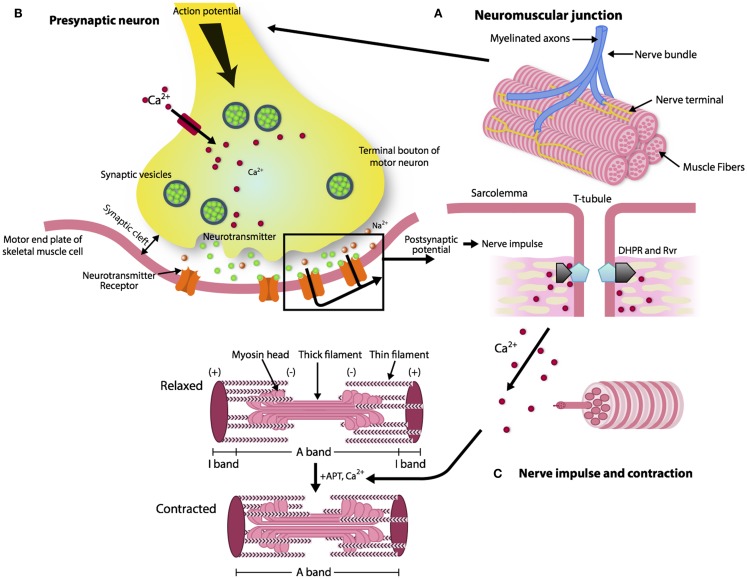
**The architecture of a neuromuscular junction (NMJ)**. **(A, B)** The NMJ is composed of three elements: pre-synaptic (motor nerve terminal), intrasynaptic (synaptic basal lamina), and post-synaptic component (muscle fiber and muscle membrane) (Punga and Ruegg, 2012). When the action potential reaches the motor nerve terminal the calcium channels open and the calcium enters in the neuron and delivers ACh in the synaptic cleft. **(C)** AChR activates the DHPRs located in the sarcolemma and by induction the RyRs. Calcium released from the sarcoplasmic reticulum through the RyRs binds to troponin C and allows cross-bridge cycling and force production.

The reason for a progressive impairment of the re-innervation process with aging is unknown, but some lines of evidence point to changes that occur with aging in the neuromuscular junction (NMJ), which is the synaptic interface between a branch of a motor neuron and muscle cells. The NMJ is composed of three elements: pre-synaptic (motor nerve terminal), intrasynaptic (synaptic basal lamina), and post-synaptic part (muscle fiber and muscle membrane) (Punga and Ruegg, 2012). When an action potential reaches the pre-synaptic element, voltage-dependent calcium channels open allowing calcium to enter the neuron and trigger the delivery of acetylcholine (ACh) in the synaptic cleft. Acetylcholine triggers nicotin acetylcholine receptors (nAChR) located in the post-synaptic membrane to produce an action potential, which in turn, activates voltage-gated dihydropyridine receptors (DHPRs) located in the sarcolemma and by induction, ryanodine receptors (RyRs). Of note, the post-synaptic membrane presents folds that expand its area. Calcium released from the sarcoplasmic reticulum through the RyRs binds to troponin C and allows cross-bridge cycling and force production (Figure 1).

Over the last decade, age-associated degeneration of the NMJ has been reported. It has been proposed that such changes may be causally related to the decline in muscle mass and function that occurs in most aging individuals. However, whether changes in the NMJ precede or follow the decline of muscle mass and strength remains unresolved. Understanding whether the primary event in the pathway to sarcopenia is muscle denervation, NMJ fragmentation or muscle fiber degeneration is important because the identification of the source of the primary event influences strategies to delay the onset of age-related muscle dysfunction. In this report, we review our current understanding of the events that lead to NMJ dysfunction with aging, including studies on biomarkers, signaling pathways, and animal models. We propose that interventions aimed at preventing the deterioration of the NMJ should be aimed at reversing the mechanisms that lead to NMJ degeneration with aging. It is important to underline that our comprehension of the global mechanism that lead to NJM impairment with aging is still patchy. Some of the elements emerging in the literature will be described and their relationship with aging explored. However, it is worth noticing that how these different parts participate and interact within a unique global mechanism and cause NMJ dysfunction with aging is not understood.

## The Aged Neuromuscular Junction

### Changes that occur with aging in the NMJ

**Figure 2:**
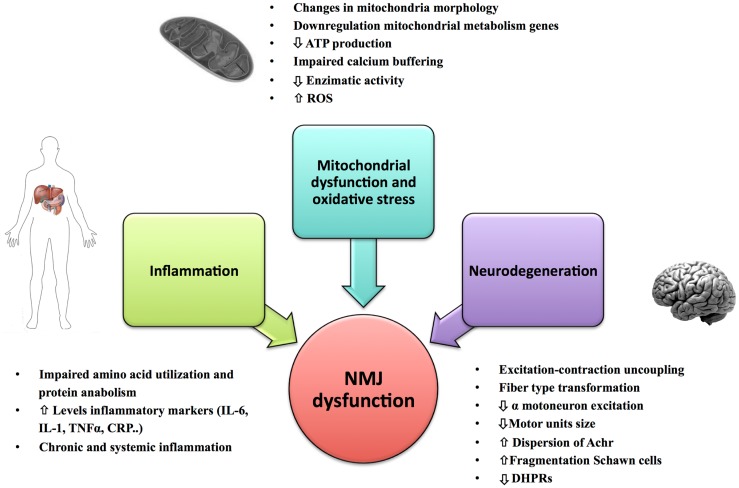
**Primary causes of NMJ dysfunction during aging**. Factors such as mitochondrial dysfunction, oxidative stress, inflammation, and changes in the innervation of muscle fiber and mechanical properties of the motor units could play an important role in the NMJ degeneration and development of sarcopenia.

**Figure 3:**
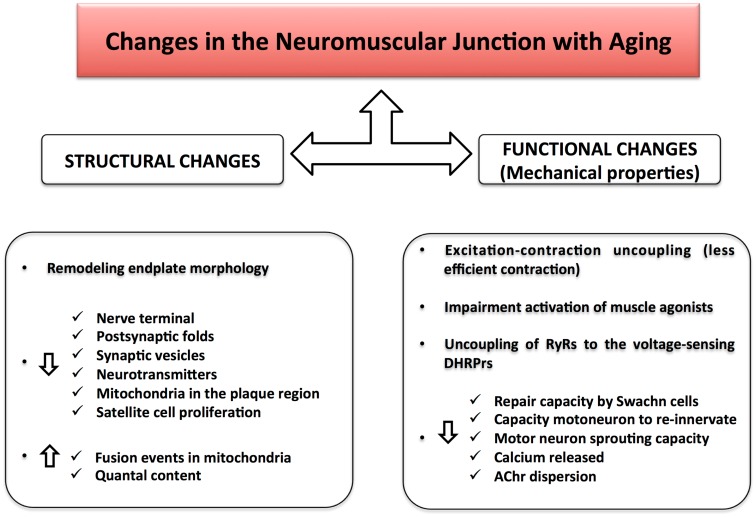
**Changes in the neuromuscular junction with aging**. Structural changes together with functional alterations result in a NMJ impairment during aging. The excitation–contraction uncoupling leads to a loss of communication between the nervous and muscular system, causing a decline in skeletal muscle strength and muscle mass.

The structure of the NMJ varies depending on the muscle fiber innervated and potentially on the level of muscle activity (Smith and Rosenheimer, 1982; Rosenheimer and Smith, 1985; Arrowsmith, 2007). There is strong evidence that changes in endplate morphology and NMJ remodeling occur with aging and precede loss of fast motor units. Morphologically, both the nerve terminal area and the number of post-synaptic folds are reduced leading to a functional impairment in the post-synaptic response of the NMJ (i.e., motor nerve conduction velocity becomes slower and the amplitude of compound muscle action potential (CMAP) decreases) (Kurokawa et al., 1999). Mitochondria in the plaque region are numerically reduced and tend to show signs of degeneration. In particular, some authors have described dramatic alterations in mitochondrial morphology in axon terminals, including cristae disruption, swelling, and formation of megamitochondria due to multiple fusions between adjacent mitochondria (Garcia et al., 2013). Studies of pre-synaptic plaque changes with aging have found high levels of oxidative damage, decreased number of synaptic vesicles, and lower quantities of neurotransmitter released during depolarization (Figure 2). These changes have been correlated with denervation of muscle fibers and muscle atrophy occurring in a fiber dependent manner (Banker et al., 1983; Jang and Van Remmen, 2011; Rowan et al., 2012). Hypothesized mechanisms include (1) progressively reduced capacity of motor neurons to re-innervate muscle fibers that are denervated or regenerating; (2) impaired excitation–contraction coupling; and (3) age-associated decline in satellite cell proliferation (Clark and Fielding, 2012; Rosso et al., 2013) (Figure 3). In particular, Clark and Fielding (2012) suggest that the NMJ activation of muscle agonists is impaired in some older adults in whom weakness is more prominent than reduced muscle mass.

As mentioned previously, some studies (Li et al., 2011) suggest that in mice, NMJ changes follow degeneration and regeneration of individual muscle fibers, while others suggest that muscle denervation might be a consequence of high energetic demand in skeletal muscle fibers, as found in human mutant SOD1 (mSOD1) mice who develop amyotrophy and muscle denervation (Dupuis et al., 2004, 2009, 2011).

#### Mitochondrial dysfunction and oxidative stress in NMJ dysfunction

Mitochondria play a critical role in regulating energy production, metabolism, signal transduction, and apoptosis and are also the primary source of oxygen free radicals generated by the dislocation of electrons traveling across the respiratory chain (Peterson et al., 2012). The numerous mitochondria in the axon not only provide energy but also buffer the large calcium ion loads which are essential for excite–contraction coupling (Barrett et al., 2011). Decline in number and function of mitochondria as well as frequent modification of their morphological structure with aging has been described in many tissues, including skeletal muscle. Dysfunctional mitochondria are often found with aging, characterized by increased levels of oxidation and nitrosylation products and decreased enzymatic activity. It has been hypothesized that the diffusion of mitochondrial nitric oxide (NO) and hydrogen peroxide (H_2_O_2_) to the cytosol is decreased in the aged brain and may impair mitochondrial biogenesis (Navarro and Boveris, 2009). Since neurons and muscle fibers are highly metabolically active, it is rational to hypothesize that they are affected by even minor mitochondrial dysfunction (Li et al., 2013; Baines et al., 2014). Indeed, it has been recently shown in sarcopenic rats with NMJ disruption that the expression of genes implicated in mitochondrial energy metabolism is down regulated (Ibebunjo et al., 2013). However, the role of oxidative stress in peripheral nervous system aging and pathology remains poorly understood (Lin and Beal, 2006; Garcia et al., 2013). Reduced ATP production and impaired calcium buffering in the subsarcolemmal mitochondria located near the NMJ may impair both neurotransmission and vesicular recycling (Deschenes, 2011).

#### Changes in the innervation of muscle fibers and motor units

Neuron loss (cell death) that occurs with aging is progressive and, as far as we know, irreversible. Loss of motor units in young adults results in re-innervation of denervated fibers by sprouting from other motor neurons and in death of fibers that are not successfully re-innervated. The details of this process have been studied mostly in animal models. Chai et al. (2011) have shown that in mice, while the size and number of alpha motor neurons in the spinal cord do not change until very late in life, profound earlier change is observed at the NMJ. In very late life, motor neurons show impaired capacity to sprout and re-innervate denervated fibers, and as a consequence, larger motor units become smaller, and more fatigable and there is a considerable atrophy of muscle fibers (Luff, 1998; Roubenoff, 2000; Glass and Roubenoff, 2010). Macroscopic age-associated muscle atrophy is due to a combination of individual fiber atrophy and a decrease in the total number of type II fibers (fast and glycolytic muscle fibers) (Lexell, 1995, 1997; Andersen, 2003; Suetta et al., 2012). These alterations in morphologic remodeling in the aged NMJ result in more dispersed AChR, with greater spatial uncoupling between ACh vesicle clusters and receptor clusters. These events together induce physiological adaptations (i.e., changes of a quantal level) in the aged NMJ that have been extensively reviewed by Deschenes (2011).

According to current views, Schwann cells (cells that myelinate axons) play an critical role in synaptic repair following denervation, through their ability to direct axonal regrowth, re-myelinate, and allow functional recovery by secreting trophic and growth factors (Rangaraju et al., 2009; Rangaraju and Notterpek, 2011; Kim et al., 2013). Impairment in these cells, such as increased fragmentation, damage, or denervation may contribute to ineffective re-innervation and neuromuscular dysfunction in aging (Verge et al., 1996; Kawabuchi et al., 2001, 2011; Gordon et al., 2009).

Changes that occur at the NMJ with aging are paralleled by a progressive uncoupling of the excitation–contraction in the skeletal muscle (Delbono et al., 1995, 1997; Delbono, 2003). It has been hypothesized that age-related muscle uncoupling is due to a mismatch between DHPR and RyR. In particular RyR would be more numerous than DHPR. This uncoupling reduces calcium release after an action potential, resulting in less efficient contraction (Wang et al., 2004; Shear and Martyn, 2009). It has been shown that insulin-like growth factor-I (IGF-1) could prevent the age-related decline in the number of DHPRs, therefore preventing the changes in nerve terminal and NMJ (Delbono, 2000, 2003; Zheng et al., 2002). These findings suggest that overexpression of IGF-1 may have a role in preventing muscle strength decline with aging.

#### Inflammation in aging (inflammaging)

Aging is characterized by high circulating levels of inflammatory markers such as interleukin 6 (IL-6), interleukin 1 (IL-1), tumor necrosis factor alpha (TNF-alpha), and C-reactive protein (CRP) in blood and tissues, often without a clear cause. This chronic low-grade inflammation in older persons has been defined by Franceschi et al. (2007) as “inflammaging.” Epidemiological studies have consistently demonstrated that “inflammaging” is a risk factor for accelerated decline of muscle mass and strength with aging, and that these changes in muscle performance may be a critical mediating step in the causal pathway between “inflammaging” and mobility disability (Ferrucci et al., 1999, 2005). In addition, medical conditions characterized by chronic overt inflammation often cause muscle wasting and weakness (Saini et al., 2007, 2009; Degens, 2010; Ferrucci et al., 2014). The mild pro-inflammatory state of aging may affect muscle performance and maintenance in many ways. For example, Schwann cell senescence has been associated with overexpression of IL-6, suggesting a role of inflammation in the age-related alterations in axonal regeneration (Saheb-Al-Zamani et al., 2013). Chronic inflammation down regulates the production of IGF-1 and blunts its biological activity. Inflammation is associated with impaired amino acid (Aa) utilization and protein anabolism, especially in critical periods such as after a meal or after a bout of exercise.

## Biomarkers of NMJ in Aging and Signaling Pathways

The direct study of the NMJ in human beings presents challenges that are almost insurmountable. The NMJ is seldom detected in muscle needle biopsies and requires open surgery biopsies that are only done for diagnostic purposes. Newly emerging neurophysiological techniques may be used in the future to study the NMJ but they are still at an early stage of development. One of the most promising areas of investigation and perhaps the one with the strongest translational potential is the study of circulating biomarkers, such as neurotrophic factors, muscle anabolic hormones, and growth factors that are known to have a role in NMJ dysfunction. Although most of the biomarkers addressed in this section are known to change with aging, whether they modulate the causal pathway from aging to NMJ impairment with aging is not fully established. Nevertheless, correlational studies may provide knowledge that in the future may help reconstructing a general model of the mechanism leading to NMJ degeneration with aging.

### Neurotrophic factors

Neurotrophins are a family of proteins implicated in neural development, maintenance, and maturation that also play a role in neurotransmission. Changes in production and response to neurotrophins with aging may contribute to reduced axonal regeneration, and dysfunction at multiple levels, including motor axons, post-synaptic membrane, and Schwann cells. One of the most studied neurotrophic factors is brain-derived neurotrophic factor (BDNF), which plays a critical role in neural plasticity and facilitates synaptic function by increasing pre-synaptic depolarization at the NMJ (Seeburger and Springer, 1993; Springer et al., 1995; Huang and Reichardt, 2001; Lipsky and Marini, 2007; Jiang et al., 2008; Gomez-Pinilla, 2011) and maintaining AChR clustering in the NMJ (Gomez-Pinilla et al., 2002; Peng et al., 2003). Interestingly, there is some initial evidence that production of glia cell-derived neurotrophic factor (GDNF) declines with aging (Li et al., 1995). Denervation leads to an up regulation of GDNF in rat and human skeletal muscle (Lie and Weis, 1998). Consistently, *in vitro* studies have found that GDNF is a potent trophic factor for motor neuron survival and a potent regulator of pre- and post-synaptic plasticity (Lin et al., 1993; Nguyen et al., 1998). Moreover, GDNF protein content in aged rat skeletal muscle might be controlled by stretching the muscle and the membrane depolarization of AChR acts to decrease GDNF protein content (McCullough et al., 2011).

There is evidence that short-term exercise increases levels of GDNF in the skeletal muscle and spinal cord of young and old rats (McCullough et al., 2013). Gyorkos et al. (2014) found increased GDNF protein levels at the end plate in the soleus and extensor digitorum longus muscles following training, supporting the idea that GDNF is activity dependent. Different models of exercise type and intensity could have varying effects on GDNF protein content in slow- and fast-twitch muscle fibers. The authors concluded that GDNF may play a role in remodeling of the NMJ in slow and fast-twitch muscle fibers.

### Insulin-like growth factor 1

Insulin-like growth factor 1 is a pleiotropic growth factor with many functions, including a neutrotrophic effect, promotion of motor neuron survival, maintenance of muscle mass and strength, and protection from oxidative stress (Yuan et al., 2000; Maggio et al., 2006, 2013; Apel et al., 2010). Many studies have found that circulating IGF-1 declines with aging and such decline may contribute to NMJ degeneration and motor unit denervation (Delbono, 2003; Messi and Delbono, 2003). In accordance with this hypothesis, in mouse models, the overexpression of muscle-specific IGF-1 reverses sarcopenia (Musaro et al., 2001), prevents the age-dependent decrease in type IIB and increase in type IIA fibers (Messi and Delbono, 2003), and also leads to improved nerve regeneration by acting on axons, Schwann cells, and the NMJ. Systemic administration of IGF-1 decreased motor neuron cell death and promoted muscle re-innervation after injury in young animals, suggesting that the decline in IGF-1 with aging may impair the ability of aging animals to repair and maintain the integrity of NMJ (Vergani et al., 1998). IGF-1 sensitivity may not decrease with age, so that IGF-1 could promote regeneration after nerve injury even in older individuals. Overexpression of muscle-specific IGF-1 in mice increases the size of NMJ without substantial changes in muscle fiber size suggesting that preservation of specific force in aged animals overexpressing IGF-1 in muscle is achieved, in part, by improved motor neurons-muscle coupling (Payne et al., 2006).

### Agrin-MuSK signaling pathway

Acetylcholine receptors clustering on the post-synaptic membrane is a main event in the differentiation of NMJ. This process requires the presence of neural agrin, a basal lamina proteoglycan that activates a muscle-specific kinase (MuSK), which is essential for AChR clustering.

Agrin is transported along the axons and finally released into synaptic basal lamina where it is inactivated by cleavage from neurotrypsin, a synaptic protease, which produces a soluble 22 kDa C-terminal agrin fragment (CAF). Importantly, CAF can be easily detected and measured in human serum (Bolliger et al., 2010; Butikofer et al., 2011). The destabilization of the NMJ by proteolysis of agrin results in precocious sarcopenia (Drey et al., 2013; Hettwer et al., 2013). This is consistent with findings in experiments with transgenic mice overexpressing neurotrypsin in spinal motoneurons that shown the full sarcopenia phenotype (Butikofer et al., 2011).

### Wnt signaling pathway

There is wide evidence, both in animals models and in humans, the Wnt signaling pathway is down regulated with aging and contributes to the progressive reduction in muscle regeneration and repair capacity (Conboy and Rando, 2012). Wnt proteins are a large family (19 members in humans) of secreted glycoproteins that are highly evolutionary conserved. Wnt signaling modulates the formation and the function of synapses and is involved in maintenance and function of many tissues including muscle and nerve. Wnt deregulation produces many neurodegenerative and mental diseases (Okerlund and Cheyette, 2011; Mulligan and Cheyette, 2012; Stamatakou and Salinas, 2013).

The role of WNT signaling on the NMJ is complex. Canonical and non-canonical Wnt pathways exert opposite effects on the formation of the vertebrate NMJ (Korkut and Budnik, 2009). The non-canonical Wnt cascade has a positive role on post-synaptic development. In contrast, activation of the canonical Wnt pathway has a negative effect on NMJ formation. Thus, while the role of Wnt signaling on NMJ development and in muscle regeneration impairment with aging is well established, evidence for a direct effect of Wnt on NMJ plasticity, maintenance, and repair is lacking and requires further investigation.

### Voltage-gated sodium channel: Na_v_1.5

The Na_v_ channel gene family (Na_v_1.1–Na_v_1.9, NaVX) codes for voltage-gated sodium channels (NaChs), which are essential for the initiation and propagation of action potentials in both nerve and muscle (Young and Caldwell, 2005). Adult skeletal muscle expresses two isoforms of Nav (Nav1.4 and Nav1.5) (Rannou et al., 2011; Kraner et al., 2012). The isoform Na_v_1.5 is particularly relevant to aging because it is mainly expressed in adult muscle following denervation (Kallen et al., 1990; Morel et al., 2010). Consistently, fibers positive for tetrodotoxin resistant (TTX-R) Na_v_1.5 channels are more prevalent in muscles from old compared with young mice, suggesting a potential biomarker for muscle denervation during aging (Wang et al., 2005; Rowan et al., 2012).

### Peroxisome proliferator-activated receptor gamma coactivator 1-alpha (PGC-1α)

Peroxisome proliferator-activated receptor gamma coactivator 1-alpha (PGC-1α) is a transcription factor that promotes mitochondrial biogenesis. Studies have consistently shown that PGC-1α decline with aging and in many age-related chronic diseases suggesting that such decline may explain the progressive mitochondrial dysfunction with aging.

Recent studies using muscle-specific PGC-1α knockout and PGC-1α overexpressing mice have suggested that PGC-1α is a key protein involved in NMJ integrity, and the decline in PGC-1α may cause NMJ degeneration with aging by a mechanism that is separate from the effect of PGC-1α on mitochondrial biogenesis (Handschin et al., 2007; Wenz et al., 2009; Liang et al., 2011; Gouspillou et al., 2013). There is recent strong evidence that elevated activity of the co-activator PGC-1α in skeletal muscle contributes to the efficient pre- and post-synaptic remodeling of the NMJ (Arnold et al., 2014).

Gouspillou et al. (2013) hypothesized that the denervation and innervation cycle observed with aging is under the control of PGC-1α expression and proposed that the aging-related decline in PGC-1α may be a central mechanism promoting instability of the NMJ and consequently aging-related alterations of myofiber innervation in sarcopenia. Interestingly, the decline in muscle PGC-1α levels with aging is attenuated by caloric restriction (CR) and exercise (Derbre et al., 2012; Garcia-Valles et al., 2013).

### Protein degradation pathways: MAFbx and MuRF1

The ubiquitin–proteasome and the autophagic-lysosomal pathways are activated during disease-related muscle atrophy and, perhaps, during the development of age-related sarcopenia. The ubiquitin–proteasome system is required to remove sarcomeric proteins, either because they are damaged or in response to decline in muscle activity. Two main enzymes at the core of this system, the ubiquitin ligases muscle atrophy f-box (Atrogin1/MAFbx) and muscle ring finger-1 (MuRF1), increase significantly in muscular atrophy, in part due to enhanced expression of tumor necrosis factor alpha (TNF-α). Mice lacking both are resistant to atrophy induced by denervation (Bodine et al., 2001; Lecker et al., 2004; Nordquist et al., 2007; Furlow et al., 2013). There is some evidence that MuRF1 could play a role in NMJ control by regulating AChR lifetime, implicating a role of MuRF1 in membrane protein turnover during aging (Rudolf et al., 2013; Franke et al., 2014; Khan et al., 2014).

## The Study of NMJ in Aging: Animals Models

The NMJ is the best model to study synaptogenesis, because, at least in animals, it is experimentally accessible in a living organism. The mechanisms that lead to differentiation and maturation of the NMJ have been studied extensively while changes that occur with aging and lead to impairment of the NMJ are not fully understood. Most of the studies conducted in this field focused on early development (i.e., the KO mice for synaptic regulators such as rapsyn, agrin, MuSK, and AChR) (Yampolsky et al., 2010; Witzemann et al., 2013). Mouse models that were originally developed to study neuromuscular diseases [i.e., amyotrophic lateral sclerosis (ALS)] could be used to explore the effect of aging on the NMJ. The prototype of these models is the Sod1^-/-^ mice.

### Sod1^-/-^ mice

The homozygous deletion of Cu/Zn superoxide dismutase (Cu/Zn SOD) leads to age-dependent muscle atrophy with alterations in NMJ similar to, but more severe than aging-related muscle atrophy (Sakellariou et al., 2011, 2014). This model is of great interest because the main function of SOD is to scavenge free oxygen radicals, being the oxidative stress, one of the landmarks of aging.

The Sod1^-/-^ mice were originally created to study ALS and addresses pathological events in the spinal cord, peripheral axons, and muscle. However, the neuromuscular sarcopenic phenotypes exhibited by this mouse share several characteristics with age-related sarcopenia, namely shift from fast to slow fiber type, mitochondrial dysfunction, and increased mitochondrial ROS generation (Jang and Van Remmen, 2009, 2011). In particular, the mitochondrial dysfunction might contribute to early motor terminal death in these mice (Muller et al., 2007). Rocha et al. (2013) have shown that the Sod1^-/-^ mice undergo cycles of denervation/re-innervation by mixed NMJ populations (Sod1a, Sod1b) supporting morphological evidence for two populations of motor units in Sod1^-/-^ mice (Schaefer et al., 2005). All together these data suggest that the impairment in neuromuscular transmission follows post-synaptic changes.

### MRF4-null mice

Muscle regulatory factor 4 (MRF4) is a member of the family of myogenic transcription factors (MyoD, myogenin, and myf-5) necessary for the differentiation of skeletal muscle (Weis et al., 2000). MRF4-null mice express genes encoding nAChR subunits and contractile proteins at normal levels, but express myogenin at dramatically increased levels. These mice have normal morphology and fiber-type composition (Zhang et al., 1995) and reduced expression of the Na_v_ 1.4 NaCh (Snow et al., 2005). Denervation leads to a rapid increase of MRF4 protein in myofiber and satellite cell nuclei in all muscle fiber types, suggesting that MRF4 might mediates the earliest responses to denervation and muscle damage (Weis et al., 2000). The NMJs in these mice at older age show higher levels of synaptic vesicle glycoprotein 2B (SV2B) a marker of synaptic vesicles and a decrease in MRF4 expression, suggesting an age-related loss of vesicles in the pre-synaptic terminal of MRF4-null mice that could lead to NMJ dysfunction (Wang et al., 2011). These authors propose MRF4 as a good therapeutic target to restoring and maintaining NMJ in aging and diseases.

## Interventions to Improve NMJ Dysfunction in Aging

Caloric restriction and exercise attenuate age-related declines in most physiological systems including the neuromuscular system (McKiernan et al., 2004; Gillette-Guyonnet and Vellas, 2008; Valdez et al., 2010; Mercken et al., 2012; Chistiakov et al., 2014). It has been shown that CR, but not exercise, blunts age-related loss of motor neurons and muscle fibers (Deschenes, 2011). Aging ‘*per se*’ limits the capacity of a person to adapt to an exercise training program since muscle plasticity is reduced with aging (Degens, 2010). Because the NMJ could be impaired during aging, with a reduced capacity to adapt to fatigue, CR and exercise are candidate interventions to delay the onset of age-related NMJ dysfunction and sarcopenia.

### Caloric restriction

Caloric restriction has long been shown to be the most effective, non-genetic intervention to extend lifespan and delay the onset of age-related diseases (McCay et al., 1935; Fontana et al., 2010; Anderson and Weindruch, 2012; Gouspillou and Hepple, 2013). Valdez et al. (2010) found that CR from 4 to 24 months of age led to sparing of many NMJs in the tibialis anterior (TA) muscle in mice. The frequencies of fragmented and denervated post-synaptic sites were all significantly lower in these calorically restricted mice than in controls. The authors concluded that CR reduced the incidence of axonal atrophy and attenuated the deleterious effects of age on the structure of the NMJ. Other authors have found that dietary restriction (40% of *ad libitum* fed diet) attenuates age-associated muscle atrophy by lowering oxidative stress in Sod1^-/-^ mice and up-regulating manganese superoxide dismutase (MnSOD), the main mitochondrial antioxidant enzyme responsible for scavenging superoxide produced by the mitochondrial electron transport chain (ETC). These data suggest that the CR model may be useful to identify mechanisms and targets for interventions aimed at preservation of NMJ with aging (Jang et al., 2012). Furthermore, Mayhew et al. (1998) propose that CR preserves the mechanical properties of aging skeletal muscles in rats through an increased expression of DHPRs.

### Exercise

Voluntary physical activity declines with aging in humans and in most other animal species. The consequent reduction in fitness leads to reduced resistance to fatigue, lower muscle strength, and increased risk of frailty (Degens and Alway, 2006; Ferrucci et al., 2008; Afilalo et al., 2014). Neuromuscular fatigue (progressively impaired transduction of action potentials through the NMJ during exercise) has been identified as a factor constraining exercise intensity and duration, and a contributor to reduced response to exercise training during aging (Belluardo et al., 2001; Deschenes, 2011). A recent review highlighted the beneficial effects of exercise on the maintenance and regeneration of NMJs (Nishimune et al., 2014). Exercise induces NMJ hypertrophy and improves recovery from peripheral nerve injuries, while decreased physical activity results in NMJ degeneration and nerve terminal sprouting (Wilson and Deschenes, 2005; Nishimune et al., 2012, 2014). Clark et al. (2013) suggest that voluntary neuromuscular activation declines with advancing age, contributing to a reduction in power production, and precedes the decline of mobility function. Aging limit the capacity of NMJ to adapt to endurance training (Deschenes et al., 2010; Valdez et al., 2010). These findings suggest that in the presence of mitochondrial DNA (mtDNA) mutation induced by ROS, strength training (ST) can reverse the loss of muscle function and altered muscle morphology associated with age and can promote the normalization of dysfunctional mitochondria (Hurley et al., 2011). Voluntary running exercise begun in late middle-age is sufficient to preserve much of the endplate nerve terminal area (Cheng et al., 2013). In mice, endurance training reduced muscle fiber size in young adults even as NMJ size increased (Deschenes, 2011). Also, in mice, Fahim (1997) showed that if the exercise training is introduced during old age, the age-related expansion of NMJ is minimized and modulated at a lower level compared with sedentary controls, indicating that the mouse NMJ undergoes a process of physiological and morphological remodeling during aging, and such plasticity could be modulated differently by endurance exercise.

## Conclusion and Future Directions

Age-related NMJ dysfunction seems to be a key to understanding musculoskeletal impairment during aging. Morphological changes together with physiological alterations result in a remodeling of the motor unit and in a decline of the number of motor neurons, particularly the type II muscle fiber. These changes lead to excitation–contraction uncoupling, and a loss of communication between the nervous and muscular system, causing a decline in skeletal muscle strength and muscle mass. Despite the extensive evidence about muscle denervation in older persons, because the direct study of NMJ in humans remains extremely challenging, it remains unclear whether denervation precedes sarcopenia or vice versa. Understanding the molecular basis of NMJ dysfunction is essential and the study of biomarker is essential both to make scientific progress in this area and translating such research in search for new treatment. Not only new structural and molecular studies, but also novel animal models to clarify what really happens to this key connection between brain and muscle are needed.

## Conflict of Interest Statement

The authors declare that the research was conducted in the absence of any commercial or financial relationships that could be construed as a potential conflict of interest.

## References

[B1] Afilalo J. Alexander K. P. Mack M. J. Maurer M. S. Green P. Allen L. A. (2014). Frailty assessment in the cardiovascular care of older adults. J. Am. Coll. Cardiol. 63, 747–762 10.1016/j.jacc.2013.09.070 24291279 PMC4571179

[B2] Andersen J. L. (2003). Muscle fibre type adaptation in the elderly human muscle. Scand. J. Med. Sci. Sports 13, 40–47 10.1034/j.1600-0838.2003.00299.x 12535316

[B3] Anderson R. M. Weindruch R. (2012). The caloric restriction paradigm: implications for healthy human aging. Am. J. Hum. Biol. 24, 101–106 10.1002/ajhb.22243 22290875 PMC3705772

[B4] Apel P. J. Ma J. Callahan M. Northam C. N. Alton T. B. Sonntag W. E. (2010). Effect of locally delivered IGF-1 on nerve regeneration during aging: an experimental study in rats. Muscle Nerve 41, 335–341 10.1002/mus.21485 19802878 PMC3045758

[B5] Arnold A. S. Gill J. Christe M. Ruiz R. McGuirk S. St-Pierre J. (2014). Morphological and functional remodelling of the neuromuscular junction by skeletal muscle PGC-1alpha. Nat Commun 5, 3569 10.1038/ncomms4569 24686533 PMC4846352

[B6] Arrowsmith J. E. (2007). The neuromuscular junction. Surgery (Oxford) 25, 105–111 10.1016/j.mpsur.2007.02.001

[B7] Baines H. L. Turnbull D. M. Greaves L. C. (2014). Human stem cell aging: do mitochondrial DNA mutations have a causal role? Aging Cell 13, 201–205 10.1111/acel.12199 24382254 PMC4331785

[B8] Banker B. Q. Kelly S. S. Robbins N. (1983). Neuromuscular transmission and correlative morphology in young and old mice. J. Physiol. 339, 355–377 6310088 10.1113/jphysiol.1983.sp014721 PMC1199166

[B9] Barrett E. F. Barrett J. N. David G. (2011). Mitochondria in motor nerve terminals: function in health and in mutant superoxide dismutase 1 mouse models of familial ALS. J. Bioenerg. Biomembr. 43, 581–586 10.1007/s10863-011-9392-1 22089637 PMC3237816

[B10] Belluardo N. Westerblad H. Mudo G. Casabona A. Bruton J. Caniglia G. (2001). Neuromuscular junction disassembly and muscle fatigue in mice lacking neurotrophin 4. Mol. Cell. Neurosci. 18, 56–67 10.1006/mcne.2001.1001 11461153

[B11] Bodine S. C. Latres E. Baumhueter S. Lai V. K. Nunez L. Clarke B. A. (2001). Identification of ubiquitin ligases required for skeletal muscle atrophy. Science 294, 1704–1708 10.1126/science.1065874 11679633

[B12] Bolliger M. F. Zurlinden A. Luscher D. Butikofer L. Shakhova O. Francolini M. (2010). Specific proteolytic cleavage of agrin regulates maturation of the neuromuscular junction. J. Cell Sci. 123(Pt 22), 3944–3955 10.1242/jcs.072090 20980386

[B13] Butikofer L. Zurlinden A. Bolliger M. F. Kunz B. Sonderegger P. (2011). Destabilization of the neuromuscular junction by proteolytic cleavage of agrin results in precocious sarcopenia. FASEB J. 25, 4378–4393 10.1096/fj.11-191262 21885656

[B14] Chai R. J. Vukovic J. Dunlop S. Grounds M. D. Shavlakadze T. (2011). Striking denervation of neuromuscular junctions without lumbar motoneuron loss in geriatric mouse muscle. PLoS ONE 6:e28090 10.1371/journal.pone.0028090 22164231 PMC3229526

[B15] Cheng A. Morsch M. Murata Y. Ghazanfari N. Reddel S. W. Phillips W. D. (2013). Sequence of age-associated changes to the mouse neuromuscular junction and the protective effects of voluntary exercise. PLoS ONE 8:e67970 10.1371/journal.pone.0067970 23844140 PMC3701007

[B16] Chistiakov D. A. Sobenin I. A. Revin V. V. Orekhov A. N. Bobryshev Y. V. (2014). Mitochondrial Aging and Age-Related Dysfunction of Mitochondria. BioMed. Res. Int. 2014, 238463 10.1155/2014/238463 24818134 PMC4003832

[B17] Clark D. J. Fielding R. A. (2012). Neuromuscular contributions to age-related weakness. J Gerontol A. Biol. Sci. Med. Sci. 67, 41–47 10.1093/gerona/glr041 21415261 PMC3260482

[B18] Clark D. J. Pojednic R. M. Reid K. F. Patten C. Pasha E. P. Phillips E. M. (2013). Longitudinal decline of neuromuscular activation and power in healthy older adults. J. Gerontol. 68, 1419–1425 10.1093/gerona/glt036 23676250 PMC3805299

[B19] Conboy I. M. Rando T. A. (2012). Heterochronic parabiosis for the study of the effects of aging on stem cells and their niches. Cell Cycle (Georgetown, Tex.) 11, 2260–2267 10.4161/cc.20437 22617385 PMC3383588

[B20] Degens H. (2010). The role of systemic inflammation in age-related muscle weakness and wasting. Scand. J. Med. Sci. Sports 20, 28–38 10.1111/j.1600-0838.2009.01018.x 19804579

[B21] Degens H. Alway S. E. (2006). Control of muscle size during disuse, disease, and aging. Int. J. Sports Med. 27, 94–99 10.1055/s-2005-837571 16475053

[B22] Delbono O. (2000). Regulation of excitation contraction coupling by insulin-like growth factor-1 in aging skeletal muscle. J. Nutr. Health Aging 4, 162–164 10936903

[B23] Delbono O. (2003). Neural control of aging skeletal muscle. Aging Cell 2, 21–29 10.1046/j.1474-9728.2003.00011.x 12882331

[B24] Delbono O. O’Rourke K. S. Ettinger W. H. (1995). Excitation-calcium release uncoupling in aged single human skeletal muscle fibers. J. Memb. Biol. 148, 211–222 10.1007/BF00235039 8747553

[B25] Delbono O. Renganathan M. Messi M. L. (1997). Excitation-Ca2+ release-contraction coupling in single aged human skeletal muscle fiber. Muscle Nerve Suppl. 5, S88–S92 10.1002/(SICI)1097-4598(1997)5+<88::AID-MUS21>3.0.CO;2-U 9331393

[B26] Derbre F. Gomez-Cabrera M. C. Nascimento A. L. Sanchis-Gomar F. Martinez-Bello V. E. Tresguerres J. A. (2012). Age associated low mitochondrial biogenesis may be explained by lack of response of PGC-1alpha to exercise training. Age (Dordr) 34, 669–679 10.1007/s11357-011-9264-y 21590341 PMC3337936

[B27] Deschenes M. R. (2011). Motor unit and neuromuscular junction remodeling with aging. Curr Aging Sci 4, 209–220 10.2174/1874609811104030209 21529328

[B28] Deschenes M. R. Roby M. A. Eason M. K. Harris M. B. (2010). Remodeling of the neuromuscular junction precedes sarcopenia related alterations in myofibers. Exp. Gerontol. 45, 389–393 10.1016/j.exger.2010.03.007 20226849 PMC2854317

[B29] Drey M. Sieber C. C. Bauer J. M. Uter W. Dahinden P. Fariello R. G. (2013). C-terminal agrin fragment as a potential marker for sarcopenia caused by degeneration of the neuromuscular junction. Exp. Gerontol. 48, 76–80 10.1016/j.exger.2012.05.021 22683512

[B30] Dupuis L. Gonzalez de Aguilar J.-L. Echaniz-Laguna A. Eschbach J. Rene F. Oudart H. (2009). Muscle mitochondrial uncoupling dismantles neuromuscular junction and triggers distal degeneration of motor neurons. PLoS ONE 4:e5390 10.1371/journal.pone.0005390 19404401 PMC2671839

[B31] Dupuis L. Oudart H. Rene F. Gonzalez de Aguilar J. L. Loeffler J. P. (2004). Evidence for defective energy homeostasis in amyotrophic lateral sclerosis: benefit of a high-energy diet in a transgenic mouse model. Proc. Natl. Acad. Sci. U S A. 101, 11159–11164 10.1073/pnas.0402026101 15263088 PMC503756

[B32] Dupuis L. Pradat P. F. Ludolph A. C. Loeffler J. P. (2011). Energy metabolism in amyotrophic lateral sclerosis. Lancet Neurol. 10, 75–82 10.1016/S1474-4422(10)70224-6 21035400

[B33] Fahim M. A. (1997). Endurance exercise modulates neuromuscular junction of C57BL/6NNia aging mice. J. Appl. Physiol. 83, 59–66 9216945 10.1152/jappl.1997.83.1.59

[B34] Ferrucci L. Baroni M. Ranchelli A. Lauretani F. Maggio M. Mecocci P. (2014). Interaction between bone and muscle in older persons with mobility limitations. Curr. Pharm. Des. 20, 3178–3197 10.2174/13816128113196660690 24050165 PMC4586132

[B35] Ferrucci L. Corsi A. Lauretani F. Bandinelli S. Bartali B. Taub D. D. (2005). The origins of age-related proinflammatory state. Blood 105, 2294–2299 10.1182/blood-2004-07-2599 15572589 PMC9828256

[B36] Ferrucci L. de Cabo R. Knuth N. D. Studenski S. (2012). Of Greek heroes, wiggling worms, mighty mice, and old body builders. J Gerontol. A. Biol. Sci. Med. Sci. 67, 13–16 10.1093/gerona/glr046 22113943 PMC3260484

[B37] Ferrucci L. Giallauria F. Schlessinger D. (2008). Mapping the road to resilience: novel math for the study of frailty. Mech. Ageing Dev. 129, 677–679 10.1016/j.mad.2008.09.007 18929593 PMC2630702

[B38] Ferrucci L. Harris T. B. Guralnik J. M. Tracy R. P. Corti M. C. Cohen H. J. (1999). Serum IL-6 level and the development of disability in older persons. J. Am. Geriatr. Soc. 47, 639–646 10366160 10.1111/j.1532-5415.1999.tb01583.x

[B39] Fontana L. Partridge L. Longo V. D. (2010). Extending healthy life span – from yeast to humans. Science 328, 321–326 10.1126/science.1172539 20395504 PMC3607354

[B40] Franceschi C. Capri M. Monti D. Giunta S. Olivieri F. Sevini F. (2007). Inflammaging and anti-inflammaging: a systemic perspective on aging and longevity emerged from studies in humans. Mech Ageing Dev 128, 92–105 10.1016/j.mad.2006.11.016 17116321

[B41] Franke B. Gasch A. Rodriguez D. Chami M. Khan M. M. Rudolf R. (2014). Molecular basis for the fold organization and sarcomeric targeting of the muscle atrogin MuRF1. Open Biol. 4, 130172 10.1098/rsob.130172 24671946 PMC3971405

[B42] Furlow J. D. Watson M. L. Waddell D. S. Neff E. S. Baehr L. M. Ross A. P. (2013). Altered gene expression patterns in muscle ring finger 1 null mice during denervation- and dexamethasone-induced muscle atrophy. Physiol. Genomics 45, 1168–1185 10.1152/physiolgenomics.00022.2013 24130153 PMC3882710

[B43] Garcia M. L. Fernandez A. Solas M. T. (2013). Mitochondria, motor neurons and aging. J. Neurol. Sci. 330, 18–26 10.1016/j.jns.2013.03.019 23628465

[B44] Garcia-Valles R. Gomez-Cabrera M. C. Rodriguez-Manas L. Garcia-Garcia F. J. Diaz A. Noguera I. (2013). Life-long spontaneous exercise does not prolong lifespan but improves health span in mice. Longev. Healthspan 2, 14 10.1186/2046-2395-2-14 24472376 PMC3922914

[B45] Gillette-Guyonnet S. Vellas B. (2008). Caloric restriction and brain function. Curr. Opin. Clin. Nutr. Metab. Care 11, 686–692 10.1097/MCO.0b013e328313968f 18827571

[B46] Glass D. Roubenoff R. (2010). Recent advances in the biology and therapy of muscle wasting. Ann. N Y Acad. Sci. 1211, 25–36 10.1111/j.1749-6632.2010.05809.x 21062293

[B47] Gomez-Pinilla F. (2011). The combined effects of exercise and foods in preventing neurological and cognitive disorders. Prev. Med. 52(Suppl. 1), S75–S80 10.1016/j.ypmed.2011.01.023 21281667 PMC3258093

[B48] Gomez-Pinilla F. Ying Z. Roy R. R. Molteni R. Edgerton V. R. (2002). Voluntary exercise induces a BDNF-mediated mechanism that promotes neuroplasticity. J. Neurophysiol. 88, 2187–2195 10.1152/jn.00152.2002 12424260

[B49] Gordon T. Udina E. Verge V. M. K. de Chaves E. I. P. (2009). Brief electrical stimulation accelerates axon regeneration in the peripheral nervous system and promotes sensory axon regeneration in the central nervous system. Motor Control 13, 412–441 20014648 10.1123/mcj.13.4.412

[B50] Gouspillou G. Hepple R. T. (2013). Facts and controversies in our understanding of how caloric restriction impacts the mitochondrion. Exp. Gerontol. 48, 1075–1084 10.1016/j.exger.2013.03.004 23523973

[B51] Gouspillou G. Picard M. Godin R. Burelle Y. Hepple R. T. (2013). Role of peroxisome proliferator-activated receptor gamma coactivator 1-alpha (PGC-1alpha) in denervation-induced atrophy in aged muscle: facts and hypotheses. Longev. Healthspan 2, 13 10.1186/2046-2395-2-13 24472348 PMC3922934

[B52] Gyorkos A. M. McCullough M. J. Spitsbergen J. M. (2014). Glial cell line-derived neurotrophic factor (GDNF) expression and NMJ plasticity in skeletal muscle following endurance exercise. Neuroscience 257, 111–118 10.1016/j.neuroscience.2013.10.068 24215980 PMC3877155

[B53] Handschin C. Kobayashi Y. M. Chin S. Seale P. Campbell K. P. Spiegelman B. M. (2007). PGC-1alpha regulates the neuromuscular junction program and ameliorates Duchenne muscular dystrophy. Genes Dev. 21, 770–783 10.1101/gad.1525107 17403779 PMC1838529

[B54] Hettwer S. Dahinden P. Kucsera S. Farina C. Ahmed S. Fariello R. (2013). Elevated levels of a C-terminal agrin fragment identifies a new subset of sarcopenia patients. Exp. Gerontol. 48, 69–75 10.1016/j.exger.2012.03.002 22433628

[B55] Huang E. J. Reichardt L. F. (2001). Neurotrophins: roles in neuronal development and function. Ann. Rev. Neurosci. 24, 677–736 10.1146/annurev.neuro.24.1.677 11520916 PMC2758233

[B56] Hurley B. F. Hanson E. D. Sheaff A. K. (2011). Strength training as a countermeasure to aging muscle and chronic disease. Sports Med. 41, 289–306 10.2165/11585920-000000000-00000 21425888

[B57] Ibebunjo C. Chick J. M. Kendall T. Eash J. K. Li C. Zhang Y. (2013). Genomic and proteomic profiling reveals reduced mitochondrial function and disruption of the neuromuscular junction driving rat sarcopenia. Mol. Cel. Biol. 33, 194–212 10.1128/MCB.01036-12 23109432 PMC3554128

[B58] Jang Y. C. Liu Y. Hayworth C. R. Bhattacharya A. Lustgarten M. S. Muller F. L. (2012). Dietary restriction attenuates age-associated muscle atrophy by lowering oxidative stress in mice even in complete absence of CuZnSOD. Aging Cell 11, 770–782 10.1111/j.1474-9726.2012.00843.x 22672615 PMC3444532

[B59] Jang Y. C. Van Remmen H. (2009). The mitochondrial theory of aging: insight from transgenic and knockout mouse models. Exp. Gerontol. 44, 256–260 10.1016/j.exger.2008.12.006 19171187

[B60] Jang Y. C. Van Remmen H. (2011). Age-associated alterations of the neuromuscular junction. Exp. Gerontol. 46, 193–198 10.1016/j.exger.2010.08.029 20854887 PMC3026920

[B61] Jiang X. Tian F. Du Y. Copeland N. G. Jenkins N. A. Tessarollo L. (2008). BHLHB2 controls Bdnf promoter 4 activity and neuronal excitability. J. Neurosci. 28, 1118–1130 10.1523/JNEUROSCI.2262-07.2008 18234890 PMC6671398

[B62] Kallen R. G. Sheng Z. H. Yang J. Chen L. Q. Rogart R. B. Barchi R. L. (1990). Primary structure and expression of a sodium channel characteristic of denervated and immature rat skeletal muscle. Neuron 4, 233–242 10.1016/0896-6273(90)90098-Z 2155010

[B63] Kawabuchi M. Tan H. Wang S. (2011). Age affects reciprocal cellular interactions in neuromuscular synapses following peripheral nerve injury. Ageing Res. Rev. 10, 43–53 10.1016/j.arr.2010.10.003 20943206

[B64] Kawabuchi M. Zhou C. J. Wang S. Nakamura K. Liu W. T. Hirata K. (2001). The spatio temporal relationship among Schwann cells, axons and post-synaptic acetylcholine receptor regions during muscle reinnervation in aged rats. Anat. Rec. 264, 183–202 10.1002/ar.1159 11590595

[B65] Khan M. M. Strack S. Wild F. Hanashima A. Gasch A. Brohm K. (2014). Role of autophagy, SQSTM1, SH3GLB1, and TRIM63 in the turnover of nicotinic acetylcholine receptors. Autophagy 10, 123–136 10.4161/auto.26841 24220501 PMC4389866

[B66] Kim H. A. Mindos T. Parkinson D. B. (2013). Plastic fantastic: Schwann cells and repair of the peripheral nervous system. Stem Cells Transl. Med. 2, 553–557 10.5966/sctm.2013-0011 23817134 PMC3726134

[B67] Korkut C. Budnik V. (2009). WNTs tune up the neuromuscular junction. Nat. Rev. Neurosci. 10, 627–634 10.1038/nrn2681 19693027 PMC3499984

[B68] Kraner S. D. Novak K. R. Wang Q. Peng J. Rich M. M. (2012). Altered sodium channel-protein associations in critical illness myopathy. Skelet. Muscle 2, 17 10.1186/2044-5040-2-17 22935229 PMC3441911

[B69] Kurokawa K. Mimori Y. Tanaka E. Kohriyama T. Nakamura S. (1999). Age-related change in peripheral nerve conduction: compound muscle action potential duration and dispersion. Gerontology 45, 168–173 10.1159/000022081 10202263

[B70] Lecker S. H. Jagoe R. T. Gilbert A. Gomes M. Baracos V. Bailey J. (2004). Multiple types of skeletal muscle atrophy involve a common program of changes in gene expression. FASEB J. 18, 39–51 14718385 10.1096/fj.03-0610com

[B71] Lexell J. (1995). Human aging, muscle mass, and fiber type composition. J Gerontol. A. Biol. Sci. Med. Sci. 50 Spec No, 11–16 7493202 10.1093/gerona/50a.special_issue.11

[B72] Lexell J. (1997). Evidence for nervous system degeneration with advancing age. J. Nutr. 127, 1011S–1013S 9164286 10.1093/jn/127.5.1011S

[B73] Li H. Kumar Sharma L. Li Y. Hu P. Idowu A. Liu D. (2013). Comparative bioenergetic study of neuronal and muscle mitochondria during aging. Free Rad. Biol. Med. 63, 30–40 10.1016/j.freeradbiomed.2013.04.030 23643721 PMC3786194

[B74] Li L. Wu W. Lin L. F. Lei M. Oppenheim R. W. Houenou L. J. (1995). Rescue of adult mouse motoneurons from injury-induced cell death by glial cell line-derived neurotrophic factor. Proc. Natl. Acad. Sci.U S A 92, 9771–9775 10.1073/pnas.92.21.9771 7568215 PMC40884

[B75] Li Y. Lee Y. I. Thompson W. J. (2011). Changes in aging mouse neuromuscular junctions are explained by degeneration and regeneration of muscle fiber segments at the synapse. J. Neurosci. 31, 14910–14919 10.1523/JNEUROSCI.3590-11.2011 22016524 PMC3213690

[B76] Liang H. Ward W. F. Jang Y. C. Bhattacharya A. Bokov A. F. Li Y. (2011). PGC-1alpha protects neurons and alters disease progression in an amyotrophic lateral sclerosis mouse model. Muscle Nerve 44, 947–956 10.1002/mus.22217 22102466

[B77] Lie D. C. Weis J. (1998). GDNF expression is increased in denervated human skeletal muscle. Neurosci. Lett. 250, 87–90 10.1016/S0304-3940(98)00434-0 9697925

[B78] Lin L. F. Doherty D. H. Lile J. D. Bektesh S. Collins F. (1993). GDNF: a glial cell line-derived neurotrophic factor for midbrain dopaminergic neurons. Science 260, 1130–1132 10.1126/science.8493557 8493557

[B79] Lin M. T. Beal M. F. (2006). Mitochondrial dysfunction and oxidative stress in neurodegenerative diseases. Nature 443, 787–795 10.1038/nature05292 17051205

[B80] Lipsky R. H. Marini A. M. (2007). Brain-derived neurotrophic factor in neuronal survival and behavior-related plasticity. Ann. NY Acad. Sci 1122, 130–143 10.1196/annals.1403.009 18077569

[B81] Luff A. R. (1998). Age-associated changes in the innervation of muscle fibers and changes in the mechanical properties of motor units. Ann. N. Y. Acad. Sci. 854, 92–101 10.1111/j.1749-6632.1998.tb09895.x 9928423

[B82] Maggio M. Ble A. Ceda G. P. Metter E. J. (2006). Decline in insulin-like growth factor-I levels across adult life span in two large population studies. J. Gerontol. A Biol. Sci. Med. Sci. 61, 182–183 10.1093/gerona/61.2.182 16510863

[B83] Maggio M. De Vita F. Lauretani F. Butto V. Bondi G. Cattabiani C. (2013). IGF-1, the cross road of the nutritional, inflammatory and hormonal pathways to frailty. Nutrients 5, 4184–4205 10.3390/nu5104184 24152751 PMC3820068

[B84] Manini T. M. Hong S. L. Clark B. C. (2013). Aging and muscle: a neuron’s perspective. Curr. Opin. Clin. Nutr. Metab. Care 16, 21–26 10.1097/MCO.0b013e32835b5880 23222705 PMC3868452

[B85] Mayhew M. Renganathan M. Delbono O. (1998). Effectiveness of caloric restriction in preventing age-related changes in rat skeletal muscle. Biochem. Biophys. Res. Commun. 251, 95–99 10.1006/bbrc.1998.9438 9790913

[B86] McCay C. M. Crowell M. F. Maynard L. A. (1935). The effect of retarded growth upon the length of life span and upon the ulti- mate body size. J. Nutr. 10, 63–79 2520283

[B87] McCullough M. J. Gyorkos A. M. Spitsbergen J. M. (2013). Short-term exercise increases GDNF protein levels in the spinal cord of young and old rats. Neuroscience 240, 258–268 10.1016/j.neuroscience.2013.02.063 23500094 PMC3637874

[B88] McCullough M. J. Peplinski N. G. Kinnell K. R. Spitsbergen J. M. (2011). Glial cell line-derived neurotrophic factor protein content in rat skeletal muscle is altered by increased physical activity in vivo and in vitro. Neuroscience 174, 234–244 10.1016/j.neuroscience.2010.11.016 21081155 PMC3020237

[B89] McKiernan S. H. Bua E. McGorray J. Aiken J. (2004). Early-onset calorie restriction conserves fiber number in aging rat skeletal muscle. FASEB J. 18, 580–581 14734642 10.1096/fj.03-0667fje

[B90] Mercken E. M. Carboneau B. A. Krzysik-Walker S. M. de Cabo R. (2012). Of mice and men: the benefits of caloric restriction, exercise, and mimetics. Ageing Res. Rev. 11, 390–398 10.1016/j.arr.2011.11.005 22210414 PMC3356510

[B91] Messi M. L. Delbono O. (2003). Target-derived trophic effect on skeletal muscle innervation in senescent mice. J. Neurosci. 23, 1351–1359 12598623 10.1523/JNEUROSCI.23-04-01351.2003 PMC6742258

[B92] Morel J. Rannou F. Talarmin H. Giroux-Metges M. A. Pennec J. P. Dorange G. (2010). Sodium channel Na(V)1.5 expression is enhanced in cultured adult rat skeletal muscle fibers. J. Membr. Biol. 235, 109–119 10.1007/s00232-010-9262-5 20517693

[B93] Muller F. L. Song W. Jang Y. C. Liu Y. Sabia M. Richardson A. (2007). Denervation-induced skeletal muscle atrophy is associated with increased mitochondrial ROS production. Am. J. Physiol. Regul. Integr. Comp. Physiol. 293, R1159–R1168 10.1152/ajpregu.00767.2006 17584954

[B94] Mulligan K. A. Cheyette B. N. R. (2012). Wnt signaling in vertebrate neural development and function. J. Neuroimmune Pharmacol. 7, 774–787 10.1007/s11481-012-9404-x 23015196 PMC3518582

[B95] Musaro A. McCullagh K. Paul A. Houghton L. Dobrowolny G. Molinaro M. (2001). Localized IGF-1 transgene expression sustains hypertrophy and regeneration in senescent skeletal muscle. Nat. Genet. 27, 195–200 10.1038/84839 11175789

[B96] Navarro A. Boveris A. (2009). Brain mitochondrial dysfunction and oxidative damage in Parkinson’s disease. J. Bioenerg. Biomembr 41, 517–521 10.1007/s10863-009-9250-6 19915964

[B97] Nguyen Q. T. Parsadanian A. S. Snider W. D. Lichtman J. W. (1998). Hyperinnervation of neuromuscular junctions caused by GDNF overexpression in muscle. Science 279, 1725–1729 10.1126/science.279.5357.1725 9497292

[B98] Nishimune H. Numata T. Chen J. Aoki Y. Wang Y. Starr M. P. (2012). Active zone protein Bassoon co-localizes with presynaptic calcium channel, modifies channel function, and recovers from aging related loss by exercise. PLoS ONE 7:e38029 10.1371/journal.pone.0038029 22701595 PMC3368936

[B99] Nishimune H. Stanford J. A. Mori Y. (2014). Role of exercise in maintaining the integrity of the neuromuscular junction. Muscle & Nerve 49, 315–324 10.1002/mus.24095 24122772 PMC4086464

[B100] Nordquist J. Hoglund A. S. Norman H. Tang X. Dworkin B. Larsson L. (2007). Transcription factors in muscle atrophy caused by blocked neuromuscular transmission and muscle unloading in rats. Mol. Med. 13, 461–470 10.2119/2006-00066 17622304 PMC2014727

[B101] Okerlund N. D. Cheyette B. N. (2011). Synaptic Wnt signaling; a contributor to major psychiatric disorders? J. Neurodev. Disord. 3, 162–174 10.1007/s11689-011-9083-6 21533542 PMC3180925

[B102] Payne A. M. Zheng Z. Messi M. L. Milligan C. E. Gonzalez E. Delbono O. (2006). Motor neurone targeting of IGF-1 prevents specific force decline in ageing mouse muscle. J. Physiol. 570(Pt 2), 283–294 10.1113/jphysiol.2005.100032 16293644 PMC1464304

[B103] Peng H. B. Yang J. F. Dai Z. Lee C. W. Hung H. W. Feng Z. H. (2003). Differential effects of neurotrophins and Schwann cell-derived signals on neuronal survival/growth and synaptogenesis. J. Neurosci. 23, 5050–5060 12832528 10.1523/JNEUROSCI.23-12-05050.2003 PMC6741189

[B104] Peterson C. M. Johannsen D. L. Ravussin E. (2012). Skeletal muscle mitochondria and aging: a review. J. Aging Res. 2012, 194821 10.1155/2012/194821 22888430 PMC3408651

[B105] Punga A. R. Ruegg M. A. (2012). Signaling and aging at the neuromuscular synapse: lessons learnt from neuromuscular diseases. Curr. Opin. Pharmacol. 12, 340–346 10.016/j.coph.2012.02.002 22365504

[B106] Rangaraju S. Hankins D. Madorsky I. Madorsky E. Lee W. H. Carter C. S. (2009). Molecular architecture of myelinated peripheral nerves is supported by calorie restriction with aging. Aging Cell 8, 178–191 10.1111/j.1474-9726.2009.00460.x 19239416 PMC2715941

[B107] Rangaraju S. Notterpek L. (2011). Autophagy aids membrane expansion by neuropathic Schwann cells. Autophagy 7, 238–239 10.4161/auto.7.2.14278 21135575 PMC3359469

[B108] Rannou F. Pennec J.-P. Morel J. Gueret G. Leschiera R. Droguet M. (2011). Na v1.4 and Na v1.5 are modulated differently during muscle immobilization and contractile phenotype conversion. J. Appl. Physiol. 111, 495–507 10.1152/japplphysiol.01136.2010 21596924

[B109] Reid K. F. Doros G. Clark D. J. Patten C. Carabello R. J. Cloutier G. J. (2012). Muscle power failure in mobility-limited older adults: preserved single fiber function despite lower whole muscle size, quality and rate of neuromuscular activation. Eur J. Appl. Physiol. 112, 2289–2301 10.1007/s00421-011-2200-0 22005960 PMC3394542

[B110] Reid K. F. Pasha E. Doros G. Clark D. J. Patten C. Phillips E. M. (2014). Longitudinal decline of lower extremity muscle power in healthy and mobility-limited older adults: influence of muscle mass, strength, composition, neuromuscular activation and single fiber contractile properties. Eur. J. Appl. Physiol. 114, 29–39 10.1007/s00421-013-2728-2 24122149 PMC3945182

[B111] Rocha M. C. Pousinha P. A. Correia A. M. Sebastiao A. M. Ribeiro J. A. (2013). Early changes of neuromuscular transmission in the SOD1(G93A) mice model of ALS start long before motor symptoms onset. PLoS ONE 8:e73846 10.1371/journal.pone.0073846 24040091 PMC3764017

[B112] Rosenheimer J. L. Smith D. O. (1985). Differential changes in the end-plate architecture of functionally diverse muscles during aging. J. Neurophysiol. 53, 1567–1581 4009233 10.1152/jn.1985.53.6.1567

[B113] Rosso A. L. Studenski S. A. Chen W. G. Aizenstein H. J. Alexander N. B. Bennett D. A. (2013). Aging, the central nervous system, and mobility. J Gerontol. A. Biol. Sci. Med. Sci. 68, 1379–1386 10.1093/gerona/glt089 23843270 PMC3805295

[B114] Roubenoff R. (2000). Sarcopenia and its implications for the elderly. Eur. J. Clin. Nutr. 54(Suppl. 3), S40–S47 10.1038/sj.ejcn.1601024 11041074

[B115] Rowan S. L. Rygiel K. Purves-Smith F. M. Solbak N. M. Turnbull D. M. Hepple R. T. (2012). Denervation causes fiber atrophy and myosin heavy chain co-expression in senescent skeletal muscle. PLoS ONE 7:e29082 10.1371/journal.pone.0029082 22235261 PMC3250397

[B116] Rudolf R. Bogomolovas J. Strack S. Choi K. R. Khan M. M. Wagner A. (2013). Regulation of nicotinic acetylcholine receptor turnover by MuRF1 connects muscle activity to endo/lysosomal and atrophy pathways. Age (Dordr) 35, 1663–1674 10.1007/s11357-012-9468-9 22956146 PMC3776120

[B117] Russ D. W. Lanza I. R. (2011). The impact of old age on skeletal muscle energetics: supply and demand. Curr. Aging Sci. 4, 234–247 10.2174/1874609811104030234 21529319

[B118] Saheb-Al-Zamani M. Yan Y. Farber S. J. Hunter D. A. Newton P. Wood M. D. (2013). Limited regeneration in long acellular nerve allografts is associated with increased Schwann cell senescence. Exp. Neurol. 247, 165–177 10.1016/j.expneurol.2013.04.011 23644284 PMC3863361

[B119] Saini A. Faulkner S. Al-Shanti N. Stewart C. (2009). Powerful signals for weak muscles. Ageing Res. Rev. 8, 251–267 10.1016/j.arr.2009.02.001 19716529

[B120] Saini A. Nasser A. S. Stewart C. E. (2007). Waste management – cytokines, growth factors and cachexia. Cytokine Growth Factor Rev. 17, 475–486 10.1016/j.cytogfr.2006.09.006 17118696

[B121] Sakellariou G. K. Davis C. S. Shi Y. Ivannikov M. V. Zhang Y. Vasilaki A. (2014). Neuron-specific expression of CuZnSOD prevents the loss of muscle mass and function that occurs in homozygous CuZnSOD-knockout mice. FASEB J. 28, 1666–1681 10.1096/fj.13-240390 24378874 PMC3963022

[B122] Sakellariou G. K. Pye D. Vasilaki A. Zibrik L. Palomero J. Kabayo T. (2011). Role of superoxide-nitric oxide interactions in the accelerated age-related loss of muscle mass in mice lacking Cu,Zn superoxide dismutase. Aging Cell 10, 749–760 10.1111/j.1474-9726.2011.00709.x 21443684 PMC3531889

[B123] Schaefer A. M. Sanes J. R. Lichtman J. W. (2005). A compensatory subpopulation of motor neurons in a mouse model of amyotrophic lateral sclerosis. J. Comparat. Neurol. 490, 209–219 10.1002/cne.20620 16082680

[B124] Seeburger J. L. Springer J. E. (1993). Experimental rationale for the therapeutic use of neurotrophins in amyotrophic lateral sclerosis. Exp. Neurol. 124, 64–72 10.1006/exnr.1993.1176 8282083

[B125] Shear T. D. Martyn J. A. J. (2009). Physiology and biology of neuromuscular transmission in health and disease. J. Crit. Care 24, 5–10 10.1016/j.jcrc.2008.08.002 19272533

[B126] Smith D. O. Rosenheimer J. L. (1982). Decreased sprouting and degeneration of nerve terminals of active muscles in aged rats. J. Neurophysiol. 48, 100–109 7119839 10.1152/jn.1982.48.1.100

[B127] Snow L. M. McLoon L. K. Thompson L. V. (2005). Adult and developmental myosin heavy chain isoforms in soleus muscle of aging Fischer Brown Norway rat. Anat. Rec. A. Discov. Mol. Cell. Evol. Biol. 286, 866–873 10.1002/ar.a.20218 16086433

[B128] Springer J. E. Seeburger J. L. He J. Gabrea A. Blankenhorn E. P. Bergman L. W. (1995). cDNA sequence and differential mRNA regulation of two forms of glial cell line-derived neurotrophic factor in Schwann cells and rat skeletal muscle. Exp. Neurol. 131, 47–52 10.1016/0014-4886(95)90006-3 7895811

[B129] Stamatakou E. Salinas P. C. (2013). Postsynaptic assembly: a role for Wnt signaling. Dev. Neurobiol. 10.1002/dneu.22138 PMC4237178 24105999

[B130] Suetta C. Frandsen U. Jensen L. Jensen M. M. Jespersen J. G. Hvid L. G. (2012). Aging affects the transcriptional regulation of human skeletal muscle disuse atrophy. PLoS ONE 7:e51238 10.1371/journal.pone.0051238 23284670 PMC3526599

[B131] Tevald M. A. Foulis S. A. Lanza I. R. Kent-Braun J. A. (2010). Lower energy cost of skeletal muscle contractions in older humans. Am. J. Physiol. Regul. Integr. Comp. Physiol. 298, R729–R739 10.1152/ajpregu.00713.2009 20032262 PMC2838655

[B132] Valdez G. Tapia J. C. Kang H. Clemenson G. D. Gage F. H. Lichtman J. W. (2010). Attenuation of age-related changes in mouse neuromuscular synapses by caloric restriction and exercise. Proc. Natl. Acad. Sci. U S A 107, 14863–14868 10.1073/pnas.1002220107 20679195 PMC2930485

[B133] Vergani L. Di Giulio A. M. Losa M. Rossoni G. Muller E. E. Gorio A. (1998). Systemic administration of insulin-like growth factor decreases motor neuron cell death and promotes muscle reinnervation. J. Neurosci. Res. 54, 840–847 10.1002/(SICI)1097-4547(19981215)54:6<840::AID-JNR12>3.3.CO;2-C 9856868

[B134] Verge V. M. Gratto K. A. Karchewski L. A. Richardson P. M. (1996). Neurotrophins and nerve injury in the adult. Philos. Trans. R. Soc. Lond. B. Biol. Sci. 351, 10.1098/rstb.1996.0038 8730781

[B135] Wang Q. Hebert S. L. Rich M. M. Kraner S. D. (2011). Loss of synaptic vesicles from neuromuscular junctions in aged MRF4-null mice. Neuroreport 22, 185–189 10.1097/WNR.0b013e328344493c 21278612 PMC3043462

[B136] Wang X. Engisch K. L. Li Y. Pinter M. J. Cope T. C. Rich M. M. (2004). Decreased synaptic activity shifts the calcium dependence of release at the mammalian neuromuscular junction in vivo. J. Neurosci. 24, 10687–10692 10.1523/JNEUROSCI.2755-04.2004 15564585 PMC6730126

[B137] Wang Z. M. Zheng Z. Messi M. L. Delbono O. (2005). Extension and magnitude of denervation in skeletal muscle from ageing mice. J. Physiol. 565(Pt 3), 757–764 10.1113/jphysiol.2005.087601 15890702 PMC1464566

[B138] Weis J. Kaussen M. Calvo S. Buonanno A. (2000). Denervation induces a rapid nuclear accumulation of MRF4 in mature myofibers. Dev. Dyn. 218, 438–451 10.1002/1097-0177(200007) 10878609

[B139] Wenz T. Rossi S. G. Rotundo R. L. Spiegelman B. M. Moraes C. T. (2009). Increased muscle PGC-1alpha expression protects from sarcopenia and metabolic disease during aging. Proc. Natl. Acad. Sci. U S A. 106, 20405–20410 10.1073/pnas.0911570106 19918075 PMC2787152

[B140] Wilson M. H. Deschenes M. R. (2005). The neuromuscular junction: anatomical features and adaptations to various forms of increased, or decreased neuromuscular activity. Int. J. Neurosci. 115, 803–828 10.1080/00207450590882172 16019575

[B141] Witzemann V. Chevessier F. Pacifici P. G. Yampolsky P. (2013). The neuromuscular junction: selective remodeling of synaptic regulators at the nerve/muscle interface. Mech. Dev. 130, 402–411 10.1016/j.mod.2012.09.004 23032192

[B142] Yampolsky P. Pacifici P. G. Witzemann V. (2010). Differential muscle-driven synaptic remodeling in the neuromuscular junction after denervation. Eur. J. Neurosci. 31, 646–658 10.1111/j.1460-9568.2010.07096.x 20148944

[B143] Young K. A. Caldwell J. H. (2005). Modulation of skeletal and cardiac voltage-gated sodium channels by calmodulin. J. Physiol. 565(Pt 2), 349–370 10.1113/jphysiol.2004.081422 15746172 PMC1464525

[B144] Yuan Q. Wu W. So K. F. Cheung A. L. Prevette D. M. Oppenheim R. W. (2000). Effects of neurotrophic factors on motoneuron survival following axonal injury in newborn rats. Neuroreport 11, 2237–2241 10.1097/00001756-200007140-00035 10923678

[B145] Zhang W. Behringer R. R. Olson E. N. (1995). Inactivation of the myogenic bHLH gene MRF4 results in up-regulation of myogenin and rib anomalies. Genes Dev. 9, 1388–1399 10.1101/gad.9.11.1388 7797078

[B146] Zheng Z. Wang Z. M. Delbono O. (2002). Insulin-like growth factor-1 increases skeletal muscle dihydropyridine receptor alpha1s transcriptional activity by acting on the camp-response element-binding protein element of the promoter region. J. Biol. Chem. 277, 50535–50542 10.1074/jbc.M210526200 12407098

